# Psychometric analysis of the Glasgow Coma Scale and its sub-scale scores in a national retrospective cohort of patients with traumatic injuries

**DOI:** 10.1371/journal.pone.0268527

**Published:** 2022-06-08

**Authors:** Bilal A. Mateen, Mike Horton, E. Diane Playford

**Affiliations:** 1 University of Warwick Medical School, Social Science and Systems in Health Unit, University of Warwick, Coventry, United Kingdom; 2 Institute of Health Informatics, University College London, London, United Kingdom; 3 The Alan Turing Institute, London, United Kingdom; 4 Psychometric Laboratory for Health Sciences, University of Leeds, Leeds, United Kingdom; University of Copenhagen, DENMARK

## Abstract

**Objectives:**

To determine the psychometric validity, using Rasch analysis, of summing the three constituent parts of the Glasgow Coma Scale (GCS).

**Design:**

National (registry-based) retrospective study.

**Setting:**

England and Wales.

**Patients:**

All individuals who sustained a traumatic injury and were: admitted for more than three days; required critical care resources; transferred for specialist management; or who died from their injuries.

**Main outcomes and measures:**

Demographic information (i.e., age at time of injury, and sex), item sub-scores of the first available GCS (either completed by the attending paramedics or on arrival to hospital), injury severity as denoted by the Injury Severity Scale (ISS), and outcome (survival to hospital discharge or 30-days post-injury, whichever is earliest).

**Results:**

321,203 cases between 2008 and 2017. 55.9% were male, the median age was 62.7 years (IQR 44.2–80.8), the median ISS was 9 (IQR 9 to 17), and 6.6% were deceased at 30 days. The reliability statistics suggest that when the extreme scores (i.e. 3 and 15) are accounted for, that there is only sufficient consistency to support the separation of injuries into 3 broad categories, e.g. mild, moderate and severe. As extreme scores don’t impact Rasch item calibrations, subsequent analysis was restricted to the 48,417 non-extreme unique cases. Overall fit to the Rasch model was poor across all analyses (p < 0.0001). Through a combination of empirical evidence and clinical reasoning, item response categories were collapsed to provide a post-hoc scoring amendment. Whilst the modifications improved the function of the individual items, there is little evidence to support them meaningfully contributing to a total score that can be interpreted on an interval scale.

**Conclusion and relevance:**

The GCS does not perform in a psychometrically robust manner in a national retrospective cohort of individuals who have experienced a traumatic injury, even after post-hoc correction.

## Introduction

Injury is a significant cause of morbidity with a global annual burden of approximately 1 billion new cases requiring some form of healthcare intervention [1], and accounting for 8% of all deaths worldwide [2]. Understanding how to stratify severity of injury and predict the risk of morbidity and mortality is an area of significant research interest [3–7]. There is one tool that has stood the test of time and continues to play a substantial role in the assessment of patients with traumatic injuries, the Glasgow Coma Scale (GCS) [8].

Since its development in 1974 [9], the GCS has been an integral part of the bedside assessment of conscious level and is widely utilized to inform clinical management as part of national and international guidelines [10–12], and for prognostication [3, 4]. However, since its introduction there has been an animated debate as to whether the three individual item sub-scale scores (Eye response, Motor response, Verbal response; more detailed description of scoring described in Table 1), should be presented either in parallel to, or in lieu of the sum of the three scores. Although early research found that the summed score appears to be (modestly) predictive of various outcomes across a range of conditions (incl. case fatality [e.g., 13, 14], functional outcome [e.g., 15, 16], cognitive outcome [e.g., 17, 18], etc.), more recently there has been increasing recognition that summing leads to information loss [19–22]. Thus, the tide appears to have shifted towards endorsing the use of the three sub-scores in lieu of the summed score. This has become especially relevant of late, given recent demonstrations that different combinations of the sub-scales with the same total score have vastly different fatality rates [23–25].

**Table 1:** The Glasgow Coma Scale.

Score	Motor (M) Responses	Verbal (V) Responses	Eye (E) Responses
6	Obey Commands		
5	Localizing to Pain	Orientated	
4	Normal Flexion	Confused	Spontaneous
3	Abnormal Flexion	Words (Inappropriate speech)	To Sound
2	Extension	Sounds (Incomprehensible speech)	To Pain
1	None	None	None

The psychometric studies to date (e.g., the illustrations of the varying predictive accuracy of different scores or permutations of the scale [23–25]) have undoubtedly been useful in identifying an underlying issue in the structure of the GCS, however, they are not the methodological tool of choice for diagnosing the nature of the problem. To understand why these results are consistently being identified, requires the use of a theory-based psychometric method, of which Rasch analysis is an example. The Rasch model [26] assumes the probability of selecting or affirming a response on a specific item (e.g., E2 or E3) depends on the patient’s underlying degree of impairment (e.g. their (decreased) conscious level), and the inherent ‘difficulty’ of that action/task (i.e. the level of impairment that is represented by the item). As such, investigating how well a pattern of observed responses conforms to the pattern predicted by the Rasch model can then be used to assess the structure and measurement properties of an outcome measurement tool [27, 28]. More in-depth discussions pertaining to the underlying mathematical model or the process of Rasch analysis can be found elsewhere [29, 30].

Using routinely collected data from the UK’s national trauma audit, the Trauma Audit Research Network (TARN), we sought to determine the psychometric validity of adding together the three constituent parts of the GCS to create a summed score, along with assessing the response structure of the three individual items (the sub-scales). Moreover, since carrying out Rasch analysis results in a post-hoc modified version of the scale in question, we sought to determine if there was any difference between the Rasch-modified version and the original GCS in predicting 30-day all-cause mortality.

## Methods

### Data source

The Trauma Audit and Research Network (TARN) is a national data collection programme. Hospitals in England and Wales submit data to TARN describing all patients who sustain a traumatic injury and are either: 1) admitted for more than three days; 2) require critical care resources, or; 3) are transferred for specialist management; or who die from their injuries [31]. TARN hosts a range of information about each case episode, but for this study we concerned ourselves only with the basic demographic information (i.e. age at time of traumatic injury, and sex), item sub-scores of the first available Glasgow Coma Scale (See Table 1; [9]), injury severity as denoted by the Injury Severity Scale (ISS) [32], and outcome (survival to hospital discharge or 30-days post-injury, whichever is earliest). GCS values are the earliest emergency department recordings where available, or if missing, then the pre-hospital score was included.

### Participants

All data pertaining to adults (18+ years) between 2008 and the beginning of 2018. The sole exclusion criteria was an incomplete GCS (not including those with a reported summed score of 3 or 15 where the breakdown could be inferred). All such instances were case-wise removed from the analysis dataset.

### Descriptive statistics

We generated and reported descriptive summaries (e.g. medians, ranges, counts, proportions, as appropriate) for the demographics, ISS, GCS sub-scale and total scores. Graphical representation and exploration of the relationship between the GCS scores and outcome for several sub-populations of this dataset have been published previously [23], and as such we have not duplicated those results here as they are not pertinent to the Rasch analysis.

### Rasch analysis

In the analysis described below, we utilized the unrestricted (partial credit) model in RUMM 2030 program [33]. Due to the extremely skewed distribution of the sample, it was not technically possible to run the analysis on the entire data set. All individuals with a minimal or maximal total score (3 or 15) were therefore excluded as these (extreme scores) do not alter the item calibrations. Extreme responses are never included in the item calibration calculations as they do not contain any useful information to measurement, so removing all extreme responses beforehand tailors the analysis to the sample where measurement is more relevant.

Moreover, although a larger sample size provides more stable item calibrations, it is known that large sample sizes overpower the fit statistics in RUMM, meaning that the logical interpretation of tests of fit and the identification of anomalies is not possible [34]. In order to address this issue, a random sample of n = 500 was drawn to assess alongside the complete dataset. A sample size of 500 provides stable item calibrations whilst providing interpretable indices [34].

#### Modelling assumptions

Rasch Analysis assumes that a set of items represent a single unidimensional construct, i.e. there is only a single factor being measured [27]. In this case, the construct represented by the GCS items would be ‘level of consciousness’. As there are only three items within the GCS, the test of unidimensionality available within RUMM 2030 is inappropriate as it is underpowered. However, any anomalies should still be identified by the other tests of fit.

A range of fit statistics are available to evaluate how well an instrument fits the Rasch model, and these are described elsewhere [29, 30, 35–38]. In this study, the overall scale fit is summarized using a χ^2^ item-trait interaction statistic, where acceptable fit is described as a non-significant χ^2^ probability value, which for this study was set at the 5% level (p = 0.05) [27, 28]. Additionally, individual item-fit statistics and psychometric characteristics were examined in order to assess the functionality of the three items, and whether they should be combined into to a single total score.

#### Reliability

The primary reliability statistic reported is the person separation index (PSI), which illustrates the ability of the GCS to differentiate between individuals with different levels of consciousness [30, 39]. A result in excess of 0.7 is deemed sufficient to be able to differentiate at least two patient groups [40]. Alternatively, a PSI value can be viewed as the proportion of instances that two randomly-selected people from the target population would be placed in the correct order by the items of the scale. E.g., A PSI value of 0.7 would order the two people correctly 70% of the time [41]. The second reliability statistic is the Cronbach’s α, where the minimum acceptable value is also 0.7 [42].

#### Threshold ordering

When an item is presented for response, it is assumed that each of the available response options represents a distinct increasing (or decreasing) level of the underlying construct. At different levels of the underlying trait, the probability of selecting a particular response will vary, with people at higher levels of the underlying construct being more likely to select a higher response category, and vice versa. The point at which there is equal probability of an individual selecting, or being classified into, two adjacent categories (e.g., E2 versus E3) are known as thresholds [27]. The theoretical probability distribution curves are available within RUMM 2030, and one manifestation of a discrepancy between the observed response pattern and the pattern predicted by the Rasch model occurs when thresholds become reversed, or disordered. This situation arises when a particular response category does not emerge as the most likely response at any point along the underlying construct. The probability of this response category being selected always remains below the likelihood of selecting alternative response options, and therefore the thresholds of the response categories become disordered (e.g., selection of the GCS E3 domain appears random, and instead the model suggests there is a tendency to go from E2 to E4). When disordered thresholds are apparent, it is an indication that the original response categories are not working as intended, possibly due to semantics, or a genuine lack of a distinct difference between the response options. A post-hoc adjustment can be made to address this issue, where adjacent responses in an item can be collapsed to produce a single new category, and the outcome of this change can be monitored within the analysis framework. In this study, we determine the optimal rescoring pattern based solely on the knowledge of which thresholds were disordered, in combination with the content of the affected response categories (i.e., selecting the most clinically relevant combination of response categories, based on their content coverage). As a sensitivity analysis, other appropriate combinations were also tested to determine whether any produced better statistical results.

### Prediction modelling

Two series of prediction modelling experiments were carried out to assess whether there was any discernable information loss in predicting case-fatality using the Rasch-modified scale in comparison to that of the original. All modelling experiments were performed using the *R (v 3*.*2*.*0)* statistical software suite [43], and the *mlr (v 2*.*7)* machine learning library [44].

In the first set of experiments, two probabilistic logistic-regression based models are generated using the original and Rasch-modified versions of the 3 sub-scale scores as well as the total score, with the respect to the binary categorical target of 30-day all-cause mortality, under a 5-fold cross validation procedure. The only additional preparatory step applied was the use of case-wise deletion for instances of a missing outcome, which was utilized to create a complete dataset. Models were compared based on out-of-sample estimates of a strictly proper scoring rule for probabilistic prediction (i.e. the Brier score). The implementation of empirical standard error estimators for classification scores is described elsewhere [45], and the exact implementation for the Brier score in R be found in the supplementary material (See S1 Appendix). Logloss, another strictly proper scoring rule, is also reported. However, the modelling pipeline described is not yet fully integrated into the underlying package (MLR) and therefore the abstraction of specific terms prevents the calculation of a standard error for the logloss metric. The performance of any specific model was considered better than another if the difference was significant at 5% significance level of a Wilcoxon signed-rank test. The choice of significance test is based on both empirical and theoretical assessments suggesting it is the appropriate choice in this setting where the assumptions of a t-test are violated [46, 47].

A second series of experiments were also carried out in which all ancillary data was included (as additive effects), i.e., age, sex, total ISS score, and most severely injured region. Here a random forest algorithm was included alongside the logistic regression as it implicitly captures any potentially interesting interactions terms without direct specification. Hyperparameter tuning was carried out using a grid search for the maximum number of trees based on the following discrete values: 100, 250, 500, 1000 and 2000. All other aspects of the pipeline were similar to the first set of experiments.

The individual-level predictions resulting from the second series of experiments was then analysed in more detail. Specifically, the number of individuals with more than a 2.5%, 5% and 10% difference in predicted risk of 30-day all-cause mortality when the rescores GCS was used versus the original (for both the logistic regression and random forest based models), was calculated. Subsequently, for the group with at least a 2.5% difference in predicted risk, the results stratified by ISS, age (in 5 year bins), and most severely injured body region were plotted.

### Ethics & governance

Data utilized in this study were made available through an agreement between the University of Warwick, the university of Leeds and the University of Manchester (on behalf of TARN). The data were anonymised by TARN prior to sharing with the research team. The study was reviewed and approved by the University of Warwick Biomedical Sciences Research Ethics Committee (Reference number: REGO-2016-1857), and the need for individual consent was waived by the ethics committee as this was retrospective analysis of national audit data.

## Results

### Sample demographics

364,355 unique cases were submitted to TARN between 2008 and 2017. After exclusion of paediatric cases (< 18 years old; 22,051 instances), and those without a complete GCS (21,101 instances), this left a complete sample of n = 321,203 (Table 2). In summary, 55.9% were male, the median age was 62.7 years (IQR 44.2 to 80.8), the median ISS was 9 (IQR 9 to 17), and a total of 6.6% were deceased at 30 days. Additional details on the sample’s injury profile can be found in S1 Appendix, Table A.

**Table 2:** Response category frequencies (n) for the individual items (sub-scales) of the Glasgow Coma Scale.

		Response Category					
Statement	Total	1	2	3	4	5	6
Eye	321203	16347	2844	12122	289890		
Verbal	321203	15622	6359	3813	26261	269148	
Motor	321203	11072	1579	1626	3332	10950	292644

### Reliability

The summary fit statistics are presented in Table 3. The individual item (sub-scale) fit statistics are presented in S1 Appendix, Table B. Overall fit to the Rasch model is poor across all analyses (p < 0.0001). Across all analyses it can be seen that the Cronbach’s Alpha value remains relatively high, with a value of 0.78 for the total non-extreme sample (i.e. Sample 1) and 0.93 in random (10%) sub-sample including extremes (i.e. Sample 3). It should be noted that the value of 0.93 is artificially high due to the high number of extreme cases, where >80% of people had the same total score. The PSI values indicate that the reliability value is in fact substantially lower across all analyses. This is likely a consequence of this statistics’ ability to account for the targeting of the scale, which the Cronbach’s Alpha does not [30]. The impact of the skewed targeting and the effect of extremes can be seen in samples 3 and 4, where the PSI values with extremes included were negative, whereas once excluded the PSI becomes positive.

**Table 3:** Summary Rasch scale fit statistics across four GCS samples, pre-and post-rescoring.

					Item Fit Residual		Person Fit Residual		Overall Chi Square Interaction			Reliability	
		Analysis	Analysis number	Valid Cases (number of extremes)	Mean	SD	Mean	SD	Value	Degrees of Freedom	p	PSI (PSI with no extremes)	Alpha (Alpha with no extremes)
**Sample 1**	**Tailored (non-extreme) sample**	Initial	1a	48417 (0)	-14.79	10.24	-0.37	0.64	8411.14	15	<0.0001	0.33 (0.33)	0.78 (0.78)
		Rescored	1b	48417 (0)	-8.72	13.11	-0.35	0.71	6712.61	12	<0.0001	0.38 (0.38)	0.72 (0.72)
**Sample 2**	**Random selection of n = 500 cases from Sample 1**	Initial	2a	500 (0)	-1.55	1.03	-0.39	0.7	108.32	15	<0.0001	0.28 (0.28)	0.76 (0.76)
		Rescored	2b	500 (0)	-0.99	1.61	-0.39	0.78	73.79	15	<0.0001	0.37 (0.37)	0.72 (0.72)
**Sample 3**	**10% of full sample**	Initial	3a	4669 (27406)	-4.78	2.76	-0.37	0.68	797.9	12	<0.0001	-0.69 (0.24)	0.93 (0.76)
		Rescored	3b	4669 (27406)	-2.97	3.48	-0.36	0.72	700.05	12	<0.0001	-0.23 (0.31)	0.92 (0.70)
**Sample 4**	**Random selection of approx. n = 500 valid cases from sample 3**	Initial	4a	491 (2909)	-1.46	1.07	-0.37	0.67	103.1	15	<0.0001	-0.67 (0.27)	0.93 (0.77)
		Rescored	4b	491 (2909)	-0.85	0.69	-0.34	0.65	75.72	12	<0.0001	-0.19 (0.35)	0.93 (0.72)
		**Target Values**			**0**	**1**	**0**	**1**	**Non-significant**			**>0.7**	**>0.7**

Results are presented across four different samples: 1) the complete non-extreme sample (n = 48,417); 2) a random sample of n = 500 from sample 1, presented for interpretable fit statistics; 3) a random 10% sample (n = 32,075) of the complete (n = 321,203) sample, which is hugely skewed and has a majority of extreme scores (valid n = 4669, extremes = 27,406); 4) a random sample of approx. n = 500 valid cases from sample 3, presented for interpretable fit statistics (valid n = 491, extremes = 2909). All results are presented both with the original response structure (the ‘a’ analysis), and post rescoring (the ‘b’ analysis).

### Threshold ordering

**Fig 1:**
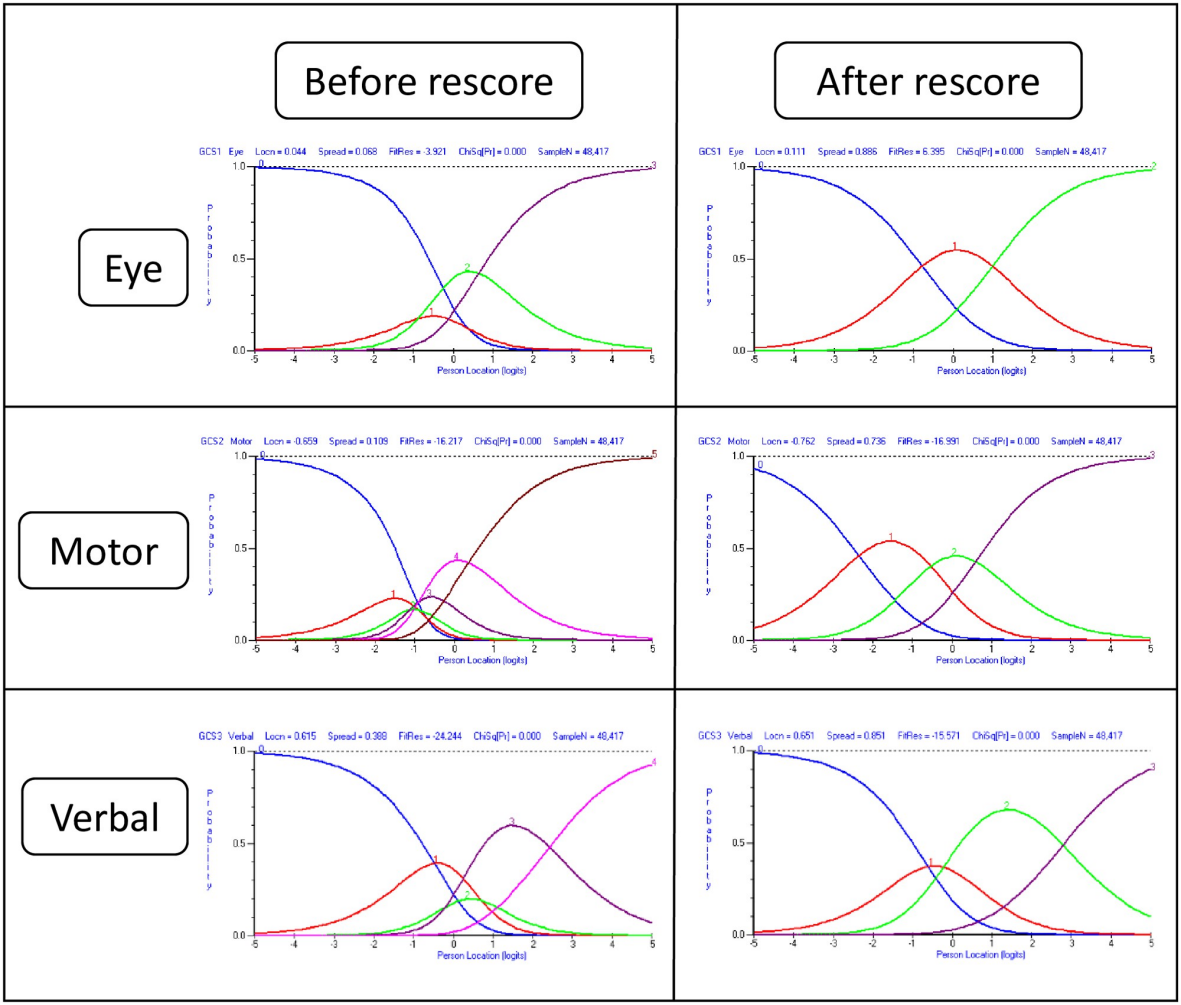
GCS response category probability distribution curves (i.e., Rasch-Andrich threshold plots), pre- and post-rescore (corresponds to sample 1a and 1b). Each plot displays person location on the ‘consciousness’ scale on the x-axis, with a higher score (to the right) representing a higher/better level. The curves represent implied probability distributions of the likelihood of a person responding in each of the response categories of the items, given their location on the scale. Prior to rescoring, the response categories were dysfunctional for all items. Post-rescore, a functional scoring system is observed.

Initially, there were disordered thresholds in all three items/sub-scales (Fig 1). This indicates that item response categories are not operating in the intended way across the continuum, meaning that the intended measurement scale is also corrupted (S1 & S2 Figs).

Through a combination of empirical evidence and clinical reasoning regarding the response category wording (carried out by authors BAM and EDP), item response categories were collapsed to provide a post-hoc scoring amendment (described in Table 4). The results for all four samples following rescoring are described in Table 3 (the ‘b’ analyses). Notably, after rescoring, the response structure clearly improves as indicated by the substantially reduced item fit residual values (Targeting plot–S3 Fig, & Item map–S4 Fig). However, in all cases there was still evidence of significant discordance with the Rasch model (illustrated by the significance of the chi-squared tests; p < 0.0001 –Table 3). The pre- and post-rescore response structure was consistent across all samples. As such only one set of Rasch-Andrich threshold plots is presented (Fig 1; corresponding to the non-extreme sample, as it is the largest). The interpretation of these results is that whilst the modifications improve the function of the individual items, there is little evidence to support them meaningfully contributing to a total score.

**Table 4:** GCS response category coding, pre- and post-rescore.

		Original response codes					
	Item	1	2	3	4	5	6
**Rescored response codes**	**Eye**	0	1		2		
	**Motor**	0	1			2	3
	**Verbal**	0	1	2		3	

### Prediction modelling with the Glasgow Coma Scale

Demographic characteristics, ISS and GCS scores for the data excluded due to absence of outcome are summarised in S1 Appendix, Table C.

Comparison of the Brier score for the logistic regression models using the original versions of the GCS sub-scale scores and the re-scored versions identified no difference in aggregate performance (Table 5). Similarly, introduction of ancillary data (i.e., age, total ISS, and most severely injured region of body based on the ISS), and a machine learning model did not result in any difference in aggregate performance when using either the original sub-scales or re-scored versions (Table 5). The logloss results are detailed in Table 6, and appear to be concordant with the aforementioned Brier score-based results.

**Table 5:** Prediction modelling (Brier score) results using the original GCS sub-scale scores compared to the re-scored versions.

Prediction modelling Experiment	Dataset utilized	Algorithm	Brier (Standard Error)	P-Value
Experiment 1 (using only GCS sub-scale data and total scores)	Original GSC	Logistic Regression	0.05 (0.001)	0.99
	Rescored GCS		0.05 (0.001)	
Experiment 2 (using the GCS and all ancillary data)	Original GSC	Logistic Regression	0.05 (0.001)	0.99
	Rescored GCS		0.05 (0.001)	
	Original GSC	Random Forest	0.04 (0.038)	0.92
	Rescored GCS		0.04 (0.029)	

**Table 6:** Prediction modelling (logloss) results using the original GCS sub-scale scores compared to the re-scored versions.

Prediction modelling Experiment	Dataset utilized	Algorithm	Logloss
Experiment 1 (using only GCS sub-scale data and total scores)	Original GSC	Logistic Regression	0.196
	Rescored GCS		0.196
Experiment 2 (using the GCS and all ancillary data)	Original GSC	Logistic Regression	0.168
		Random Forest	1.316
	Rescored GCS	Logistic Regression	0.169
		Random Forest	0.123

Further interrogation of the individual-level results from experiment 2 suggests that only a small fraction of individuals have potentially clinically significant differences in their predicted risk of 30-day all-cause mortality. For the logistic regression based model, using 2.5%, 5% and 10% as the thresholds for clinically significant difference in predicted risk, the respective number of individuals above the thresholds are: 8668 (2.83% of the sample), 3238 (1.03%), and 170 (0.06%). Stratification of the differences between the two logistic regression models’ individual-level predictions suggests that the model containing the rescored version has a systematic tendency to predict lower probabilities of 30-day all-cause mortality (S5 and S6 Figs). For the random forest models, using the same threshold, the respective number of individuals is: 15696 (5.13%), 9936 (3.25%), and 4502 (1.47%). Stratification of the individual-level results for the random forest models does not identify the same systematic bias and instead illustrates a much more symmetrical pattern (S7 and S8 Figs).

## Discussion

Using a national sample of individuals with traumatic injuries, Rasch-analysis indicates that the Glasgow Coma Scale (GCS) does not appear to function in a psychometrically robust manner. The reliability statistics (i.e. the PSI) suggest that when the extreme scores (i.e. 3 and 15) are accounted for, that there is still only sufficient consistency to support the separation of injuries into 3 broad categories based on the GCS, e.g. mild, moderate and severe [40]. Anything more detailed than this, such as case-fatality rates assigned to individual total scores should be interpreted with extreme caution (e.g., Table 7). Importantly, we do not interpret the Cronbach’s alpha because the discordance between the two measures of reliability is reflective of the aforementioned skewed targeting, and in these settings the PSI is more useful.

**Table 7:** Re-scored GCS sub-scales organized by interval logit location.

GCS Sub-scale Threshold	Proposed Language for Threshold	Mortality Rate (by most severely injured body region)*					
		Abdomen	Chest	Head	Limbs	Spine	Multiple
*Baseline score of 3 for all items (no movement*, *no verbal response or eye movement)*		71.5%	67.9%	63.5%	56.7%	69.3%	63.0%
M1	Any movement other than localizing in response to a stimulus	31.3%	25.7%	53.8%	29.8%	29.3%	32.7%
E1	Opening eyes to stimulus (sound or pain)	25.0%	20.1%	34.8%	12.5%	30.4%	24.5%
V1	Making sounds or incomprehensible speech	21.1%	22.5%	27.2%	9.3%	28.4%	13.1%
M2	Localizing to pain	12.5%	15.4%	25.6%	9.3%	25.3%	15.6%
V2	Confused or inappropriate words	18.4%	18.3%	21.2%	9.4%	20.5%	12.4%
M3	Obeys commands	8.8%	11.2%	14.6%	8.6%	16.0%	10.2%
E2	Eyes spontaneously open	4.8%	8.1%	10.9%	6.2%	8.9%	6.2%
V3	Orientated and verbalizing appropriately	1.6%	3.8%	6.8%	2.3%	3.0%	2.9%

M = motor, V = verbal, E = Eye. Note: Data utilized in this table to generate the mortality statistics is based on the cleaned modelling dataset where individuals without a recorded outcome were case-wise deleted.

*The other and face groups have not been included as the former is a heterogeneous catch-all containing several different injury phenotypes, and the latter only recorded 47 mortalities and thus the proportions have large associated uncertainties. Note the inappropriate increase in mortality risk at the following transitions: Abdomen M2 -> V2, Chest E1 -> V1 & M2 -> V2, Spine M1 -> E1, and Multiple V1 -> M2.

Post-hoc rescoring of the GCS based on a combination of empirical evidence (i.e., the Rasch-Andrich threshold plots) and the clinical relevance of the response category content appears to improve the operation of the GCS (to an extent). However, there is still no psychometric evidence to support summing the subscales into a total score (see Table 2). Moreover, the prediction modelling experiments indicate that the re-scored sub-scales contain comparable information to the original response scale, however, there is a small proportion of individuals for whom there are potentially clinically significant differences in predicted probability of mortality (at most 5% of the sample depending on the modelling approach and threshold for clinical significance). It is worth noting at this point that the GCS may well demonstrate different behaviour in predicting different outcomes, and thus, the results of this study may not be generalizable to other outcomes such as those related to morbidity.

### Context

There are several studies that explore the psychometric properties of the GCS using classical test theory-based approaches [48–50], most often using data specific to traumatic brain injuries. Importantly, the results of this study are explicitly concordant with the aforementioned contributions. For example, Reith et al. previously described how the three sub-scales of the GCS appear to have ceiling effects that arise in the order: motor, then eye, and finally verbal in three different samples of patients with traumatic brain injuries [23]. S4 Fig clearly illustrates this pattern, with the highest difficulty item on the post-hoc corrected interval version of the GCS being a verbal sub-scale specific threshold, followed by the last eye-subscale threshold, and then motor. Therefore, we confirm that the GCS behaves in this way at the top end of potential scores for all injuries, not just traumatic brain injuries as previous studies have [e.g., 23], and we have done so using an entirely different psychometric analysis methodology. Finally, we extend the current knowledge base by showing the relative position of all of the GCS sub-scale thresholds (S2 and S4 Figs). Doing so, allows us to map each score to a single case-fatality risk (Table 7). However, as stated earlier these results should be handled with caution due to the very low PSI. It has been included here as it provides insight into an area of potential future research. The abdomen, chest, spine and multiple groups all have transitions where the risk of mortality appears to rise as the rescored GCS increases. This is suggestive of systematic differences in item functioning by the most severely injured body region (also known as DIF; differential item functioning in the Rasch community). Future research might consider the need for (trauma) phenotype-specific versions of the GCS to address this issue.

### Strengths and limitations

The combination of a large national sample, multiple sensitivity analyses using different sub-sampling schemes, and a well-established item-response theory informed psychometric validation methodology (i.e. Rasch analysis) suggests that the reported results are both likely to be robust and generalizable. Moreover, the demonstration of the statistically indistinguishable performance of the different the sub-scale scoring using a methodologically robust predictive modelling framework, provides clear evidence that the rescoring scheme derived using Rasch analysis presents a meaningful way to improve how the GCS is currently utilized, by highlighting how uninformative response categories can be discarded without loss of information. However, despite these strengths there are still several limitations that are worth discussing.

One of the principal weaknesses of this study is of the use of the first recorded GCS score, without differentiation of the healthcare professional (HCPs) which carried out the assessment, or the point of the assessment. There is well documented evidence that proficiency in carrying out the GCS assessment differs between HCP groups [51], and that scores in the pre-hospital setting are often discordant to those recorded in the emergency department [52, 53]. As such, our inability to control for these differences might have introduced noise into the Rasch modelling which could (in part) explain the difficulty in getting the data to conform to the models’ expectations. Next, the use of case-wise deletion could be argued to potentially introduce selection bias, however, as with previous studies using this data we felt that an assumption of missingness at random (likely due to logistical reasons) was more apt, and thus consistent with the use of complete case analysis [23]. Moreover, we also need to consider that the items weren’t presented in the way(s) that the post-hoc corrections propose that they should—instead these are based on theoretical probability distributions. As such, this study cannot justify the use of the post-hoc corrected version in practice, which would require dedicated feasibility testing and psychometric analysis in its own right. Furthermore, a limitation of the methods employed is that although mortality is a clinically important outcome the results cannot be interpreted as general proof of the re-scored sub-scales containing similar amounts of information as the original versions as that would have required the use of an ‘entropy’ metric to demonstrate.

And finally, it is possible that the dimension of ‘consciousness’ being measured by the GCS differs based on more than just the body-part that was injured; there are numerous ways (both directly and indirectly-related to a trauma) in which consciousness can be depressed. Notably, the Rasch literature contains a rich reflection on the nature of causal and correlational observations, and how they map to measurement indicators [54–56]. As such, future research might appropriately prioritise the contextualisation of GCS scores in a more detailed profile of the individual experiencing the depressed consciousness, including any complications and pre-existing comorbidities that might be driving the observed result rather than the trauma itself.

### Implications for researchers and clinicians

Closer inspection of the statistical outputs provides several inferences which have direct clinical and academic relevance. Firstly, the aforementioned skewed targeting is problematic if the GCS is being used as an outcome measure, as the scale is unable to reliably order patients correctly, or to distinguish ‘ability’ groups between a score of 3 and 15. This is important, as it directly contradict recent suggestions by the original developers of the GCS, that the tool might be useful for describing sub-populations! [8] And secondly, the results clearly illustrate that certain thresholds in the original GCS are likely uninformative. As such, the stratification of outcomes by baseline GCS sub-scale scores commonly reported in clinical trials is presumably invalid as it separates groups (e.g. E1 and E2) that are arguably arbitrarily assigned. Re-analysis of trial results based on these observations is clearly justified.

## Conclusion

This study illustrates that the Glasgow Coma Scale (GCS) does not perform in a psychometrically robust manner in a national retrospective cohort of individuals who have experienced a traumatic injury, using a Rasch-analysis based approach. In lieu of this less-than-ideal response structure, we present a more psychometrically robust and parsimonious version of three Glasgow Coma sub-scales, which performs similarly in modelling experiments predicting 30-day all-cause mortality risk to the original version.

## Supporting information

S1 Appendix Code for implementation of Brier score and Tables A-C. (PDF)

S1 Fig Targeting plot of non-extreme sample—Original scoring (corresponds to sample 1a). The targeting plot displays the relative locations of all persons and items within the analysis on the same logit location ‘consciousness’ scale, with a higher score (to the right) representing a higher/better level.
(TIF)

S2 Fig Item map of non-extreme sample—Original scoring (corresponds to sample 1a). The Item Map displays the relative locations of all persons and items within the analysis on the same logit location ‘consciousness’ scale, with a higher score representing a higher/better level. This plot is based on Sample 1a: the original response structure of the complete non-extreme sample (n = 48,417). Item codes are as follows: GCS1 = Eye, GCS2 = Motor, GCS3 = Verbal; where .1-.5 represent the response category thresholds for each item. Note that they are disordered at this stage of the analysis.
(TIF)

S3 Fig Targeting plot of non-extreme sample post-rescore (corresponds to sample 1b). The targeting plot displays the relative locations of all persons and items within the analysis on the same logit location ‘consciousness’ scale, with a higher score (to the right) representing a higher/better level.
(TIF)

S4 Fig Item map of non-extreme sample post-rescore (corresponds to sample 1b). The Item Map displays the relative locations of all persons and items within the analysis on the same logit location ‘consciousness’ scale, with a higher score representing a higher/better level. This plot is based on Sample 1b: the rescored response structure of the complete non-extreme sample (n = 48,417). Item codes are as follows: GCS1 = Eye, GCS2 = Motor, GCS3 = Verbal; where .1-.5 represent the response category thresholds for each item. Note that they are now ordered at this stage of the analysis.
(TIF)

S5 Fig Histogram of differences in logistic regression model based predictions, stratified by most severely injured body part. Differences in the predicted probability of 30-day all-cause mortality using a logistic regression model and all ancillary variables in combination with either the original or rescored version of the GCS, stratified by the ‘most severely injured body part’ based on the Injury Severity Score. The sample used for this histogram is restricted to those with at least a +/-2.5% difference in predicted probabilities.
(TIF)

S6 Fig Box-plot of differences in logistic regression model based predictions, stratified by age and injury Severity Score. Median and IQR for differences in the predicted probability of 30-day all-cause mortality using a logistic regression model and all ancillary variables in combination with either the original or rescored version of the GCS, for binned age groups and Injury Severity Scores. The sample used for this histogram is restricted to those with at least a +/-2.5% difference in predicted probabilities.
(TIF)

S7 Fig Histogram of differences in the random forest based predictions, stratified by most severely injured body part. Differences in the predicted probability of 30-day all-cause mortality using a random forest model and all ancillary variables in combination with either the original or rescored version of the GCS, stratified by the ‘most severely injured body part’ based on the Injury Severity Score. The sample used for this histogram is restricted to those with at least a +/-2.5% difference in predicted probabilities.
(TIF)

S8 Fig Box-plot of differences in random forest based predictions, stratified by age and Injury Severity Score. Median and IQR for differences in the predicted probability of 30-day all-cause mortality using a random forest model and all ancillary variables in combination with either the original or rescored version of the GCS, for binned age groups and Injury Severity Scores. The sample used for this histogram is restricted to those with at least a +/-2.5% difference in predicted probabilities.
(TIF)
