# Supplementary material for: Psychometric analysis of the Glasgow Coma Scale and its sub-scale scores in a national retrospective cohort of patients with traumatic injuries
Source: PLoS One. 2022 Jun 8;17(6):e0268527. doi: 10.1371/journal.pone.0268527 (PMC9176762; doi:10.1371/journal.pone.0268527)
Supplement: S1 Appendix — (PDF) [file pone.0268527.s001.pdf]

Code: Implementation of the Standard Error for a Brier score in R

```
Brier.se = makeMeasure(
        id = "Brier.se", name = "Standard Error of Brier Score",
        properties = c("classif", "classif.multi", "req.pred", "req.truth"),
        minimize = TRUE, best = 0, worst = Inf,
        fun = function(task, model, pred, feats, extra.args) {
            measureBrier.SE(getPredictionProbabilities(pred), pred$data$truth,
            pred$task.desc$negative, pred$task.desc$positive)
            })

measureBrier.SE <- function(probabilities,truth,negative,positive){
                N <- length(truth)
                y <- as.numeric(positive == truth)
                p <- (y - probabilities)^2
              return(sd(p)/sqrt(N))
              }
```

Table A: Glasgow Coma Scale Total Score Response Frequencies

GCS Total Score	Frequency (Proportion)	Most Severely Injured Body Part by ISS (number of individuals with a similar GCS)								Mortality (Proportion of those with similar GCS*)
		Limbs	Head	Chest	Spine	Abdomen	Multiple	Other	Face	
3	9860 (3.1%)	278	5570	1091	360	153	1047	1318	43	6013 (61.0%)
4	1351 (0.4%)	30	1035	69	26	11	113	63	4	671 (35.9% - 55.9%)
5	1108 (0.3%)	27	824	56	21	6	107	58	9	434 (24.6% - 47.6%)
6	1516 (0.5%)	50	1097	101	31	11	165	55	6	508 (23.8% - 45.0%)
7	1894 (0.6%)	51	1495	109	23	15	151	38	12	447 (11.1% - 48.5%)
8	1938 (0.6%)	67	1446	138	53	18	175	26	15	410 (10.5% - 38.5%)
9	1772 (0.6%)	111	1240	134	49	16	171	30	21	358 (12.5% - 36.0%)
10	2383 (0.7%)	158	1672	174	77	34	208	36	24	466 (10.7% - 32.6%)
11	2662 (0.8%)	241	1710	247	100	41	262	37	24	519 (0.0% - 33.3%)
12	3114 (1.0%)	313	1956	318	146	53	278	23	27	472 (0.0% - 20.0%)
13	6632 (2.1%)	807	3889	706	409	102	633	27	59	722 (8.0% - 16.4%)
14	24047 (7.5%)	5029	10352	3083	2412	403	2397	82	289	1932 (6.0% - 8.6%)
15	262926 (81.9%)	108674	34244	44985	39083	6652	23822	861	4605	8201 (3.1%)
**Total**	**321203**	**115,836**	**66,530**	**51,211**	**42,790**	**7,515**	**29,529**	**2,654**	**5138**	**21,153 (6.6%)**

*Legend: GCS: Glasgow Coma Scale; ISS: Injury Severity Scale. *The reported mortality rates are the minimum and maximum observed for the possible combinations that result in each total score (i.e. 14 can be composed of E3 V5 M6 or E4 V4 M6 or E4 V5 M5).*

Table B: Individual item (sub-scale) fit statistics across four GCS samples, pre-and post-rescoring

Analysis	Item	Location	SE	FitResid	ChiSq	DF	Prob
1a	Eye	0.044	0.006	-3.921	3200.68	5	0.000
	Motor	-0.659	0.005	-16.217	3873.52	5	0.000
	Verbal	0.615	0.005	-24.244	1336.94	5	0.000
1b	Eye	0.111	0.008	6.395	2909.33	4	0.000
	Motor	-0.762	0.008	-16.991	3173.70	4	0.000
	Verbal	0.651	0.007	-15.571	629.58	4	0.000
2a	Eye	0.027	0.059	-0.389	43.40	5	0.000
	Motor	-0.616	0.05	-1.905	43.26	5	0.000
	Verbal	0.589	0.052	-2.359	21.66	5	0.001
2b	Eye	0.104	0.081	0.838	30.59	5	0.000
	Motor	-0.785	0.075	-2.183	36.59	5	0.000
	Verbal	0.68	0.07	-1.619	6.62	5	0.251
3a	Eye	0.014	0.019	-2.018	329.18	4	0.000
	Motor	-0.579	0.017	-4.794	357.15	4	0.000
	Verbal	0.566	0.017	-7.538	111.57	4	0.000
3b	Eye	0.068	0.026	1.049	315.78	4	0.000
	Motor	-0.697	0.024	-4.999	314.77	4	0.000
	Verbal	0.629	0.022	-4.949	69.49	4	0.000
4a	Eye	0.04	0.061	-0.916	49.38	5	0.000
	Motor	-0.562	0.051	-0.774	32.96	5	0.000
	Verbal	0.521	0.053	-2.703	20.68	5	0.001
4b	Eye	0.147	0.081	-0.066	36.22	4	0.000
	Motor	-0.656	0.075	-1.14	28.59	4	0.000
	Verbal	0.509	0.071	-1.353	10.91	4	0.028

Legend: Location = Logit location of item on Rasch-calibrated scale. SE = Standard Error of location estimate. FitResid = Z-Standardised Fit Residual. ChiSq = Chi Square value. DF = Degrees of Freedom. Prob = Probability (of result occurring by chance). Results are presented across four different samples: 1) the complete non-extreme sample (n=48,417); 2) a random sample of n=500 from sample 1, presented for interpretable fit statistics; 3) a random 10% sample (n=32,075) of the complete (n=321,203) sample, which is hugely skewed and has a majority of extreme scores (valid n=4669, extremes=27,406); 4) a random sample of approx. n=500 valid cases from sample 3, presented for interpretable fit statistics (valid n=491, extremes=2909). All results are presented both with the original response structure (the 'a' analysis), and post rescoring (the 'b' analysis).

Table C: Descriptive summaries of data excluded from the prediction modelling experiments due to missingness of outcome data.

	**Casewise excluded for absence of outcome (n = 15,052)**
**Male Sex (Proportion)**	9900 (65.8%)
**Median Age in Years (IQR)**	56.1 (37.6 – 74.3)
**Median ISS (IQR)**	16.0 (9.0 – 25.0)
**Most Severely Injured Body Part**	
Limbs	3993 (26.5%)
Head	4398 (29.2%)
Chest	1791 (11.9%)
Spine	2020 (13.4%)
Abdomen	(in Other)
Face	(in Other)
Multiple	1806 (12.0%)
Other	1044 (6.9%)
**Total GCS Score**	
3	824 (5.5%)
4	125 (0.8%)
5	96 (0.6%)
6	135 (0.9%)
7	207 (1.4%)
8	220 (1.5%)
9	149 (1.0%)
10	205 (1.4%)
11	211 (1.4%)
12	214 (1.4%)
13	450 (3.0%)
14	1321 (8.8%)
15	10895 (72.4%)
