# Supplementary figures and images for: Psychometric analysis of the Glasgow Coma Scale and its sub-scale scores in a national retrospective cohort of patients with traumatic injuries
Source: PLoS One. 2022 Jun 8;17(6):e0268527. doi: 10.1371/journal.pone.0268527 (PMC9176762; doi:10.1371/journal.pone.0268527)

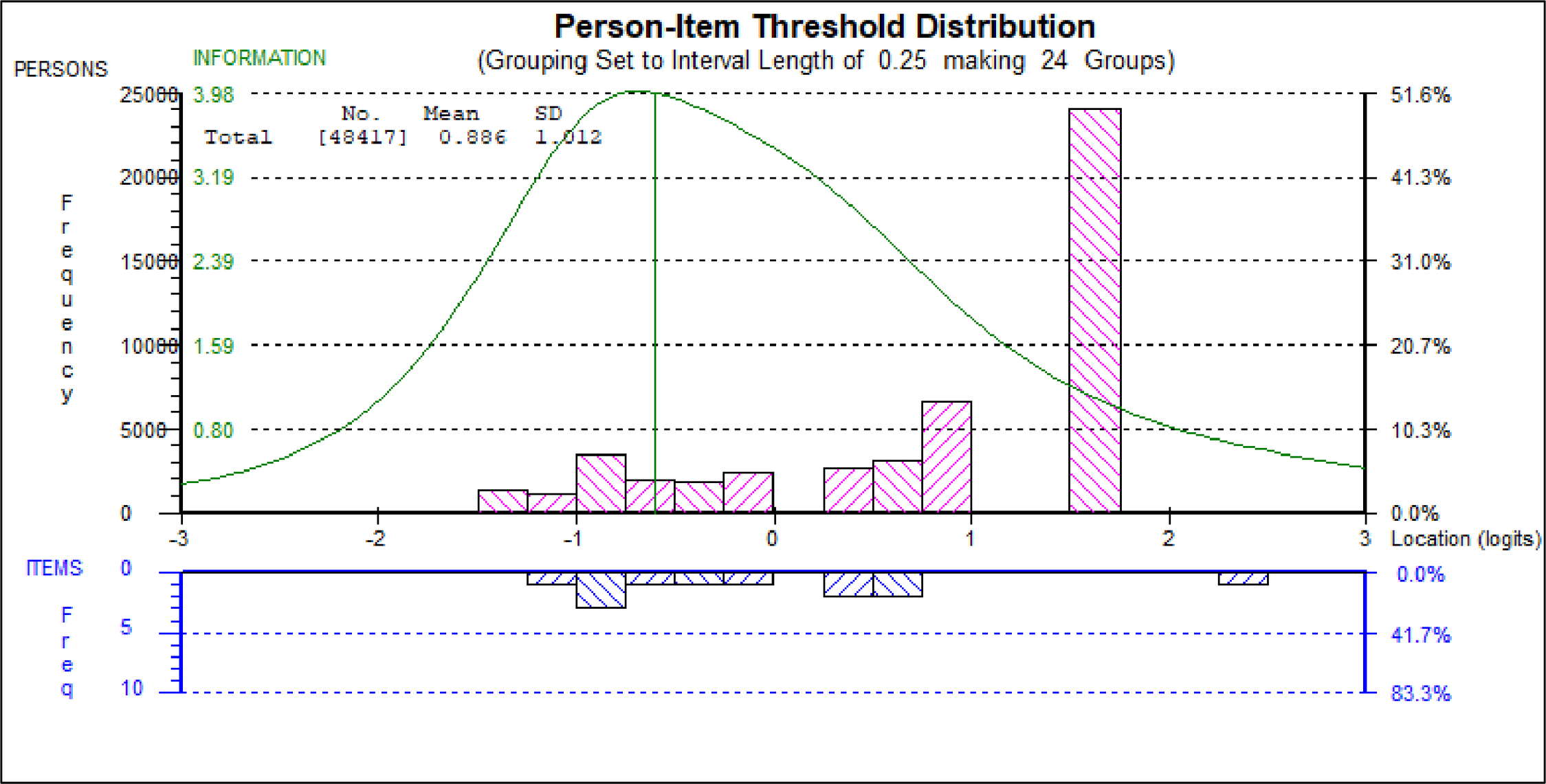

Supplement: S1 Fig — The targeting plot displays the relative locations of all persons and items within the analysis on the same logit location ‘consciousness’ scale, with a higher score (to the right) representing a higher/better level. (TIF) [file pone.0268527.s002.tif]

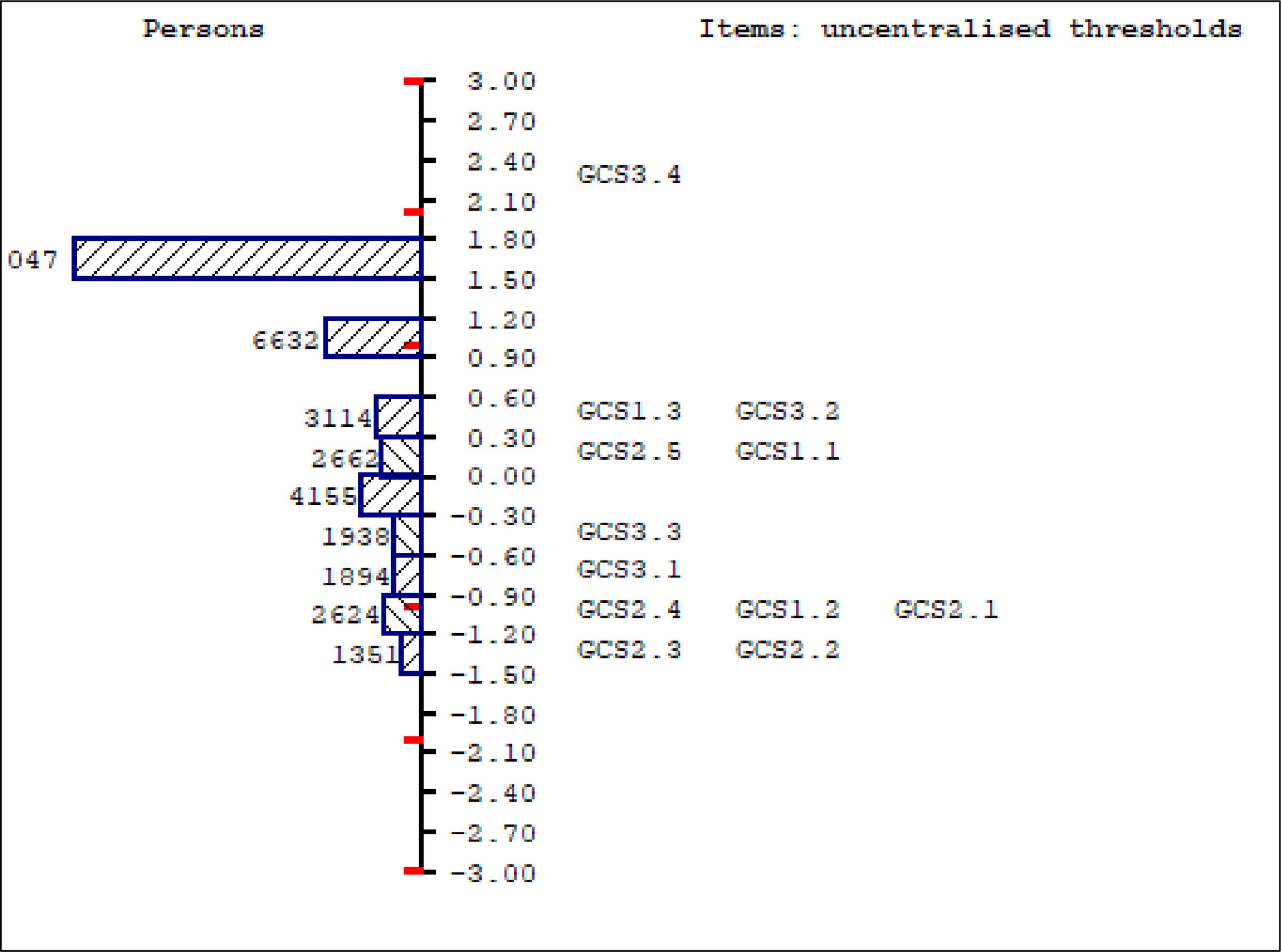

Supplement: S2 Fig — The Item Map displays the relative locations of all persons and items within the analysis on the same logit location ‘consciousness’ scale, with a higher score representing a higher/better level. This plot is based on Sample 1a: the original response structure of the complete non-extreme sample (n = 48,417). Item codes are as follows: GCS1 = Eye, GCS2 = Motor, GCS3 = Verbal; where .1-.5 represent the response category thresholds for each item. Note that they are disordered at this stage of the analysis. (TIF) [file pone.0268527.s003.tif]

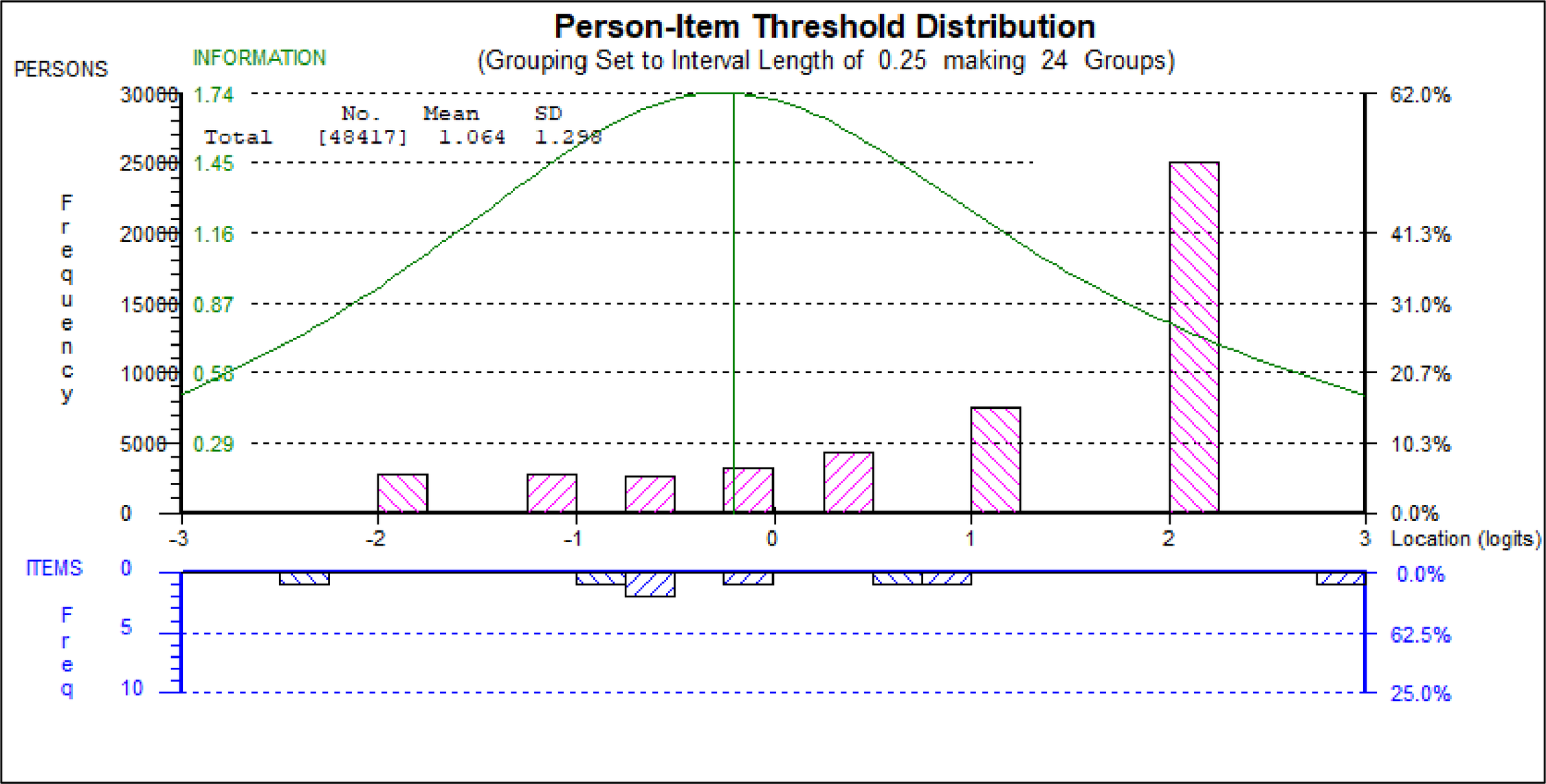

Supplement: S3 Fig — The targeting plot displays the relative locations of all persons and items within the analysis on the same logit location ‘consciousness’ scale, with a higher score (to the right) representing a higher/better level. (TIF) [file pone.0268527.s004.tif]

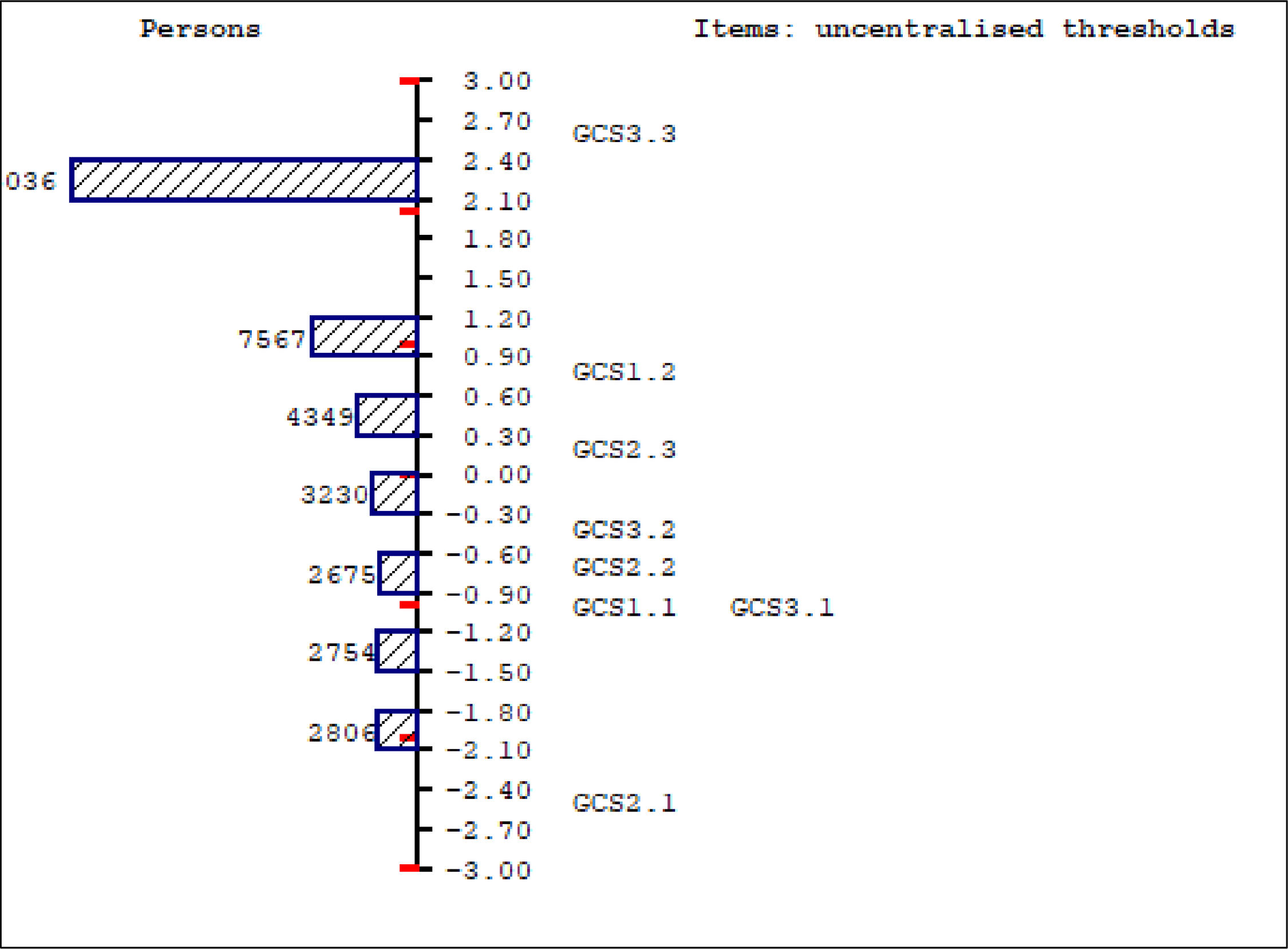

Supplement: S4 Fig — The Item Map displays the relative locations of all persons and items within the analysis on the same logit location ‘consciousness’ scale, with a higher score representing a higher/better level. This plot is based on Sample 1b: the rescored response structure of the complete non-extreme sample (n = 48,417). Item codes are as follows: GCS1 = Eye, GCS2 = Motor, GCS3 = Verbal; where .1-.5 represent the response category thresholds for each item. Note that they are now ordered at this stage of the analysis. (TIF) [file pone.0268527.s005.tif]

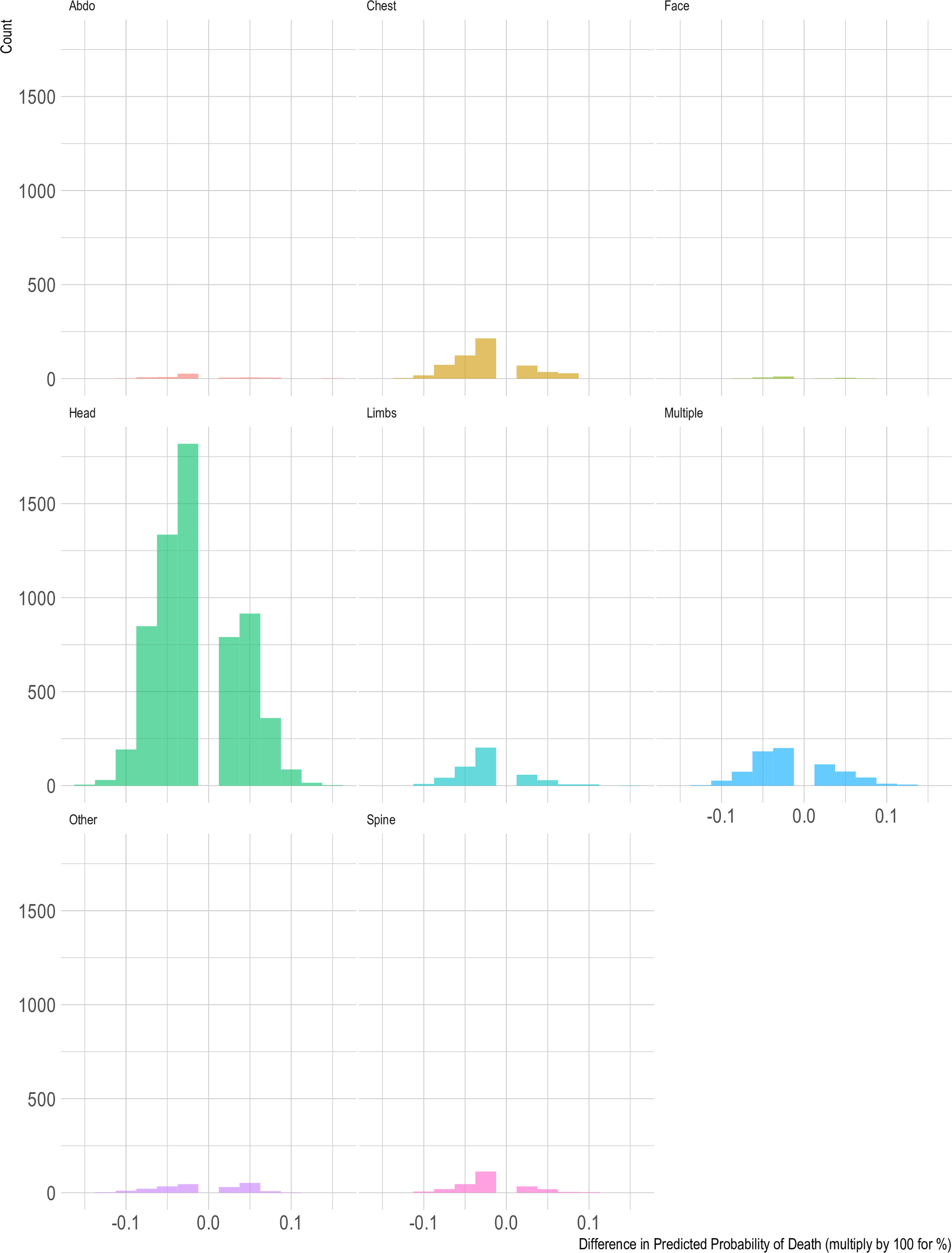

Supplement: S5 Fig — Differences in the predicted probability of 30-day all-cause mortality using a logistic regression model and all ancillary variables in combination with either the original or rescored version of the GCS, stratified by the ‘most severely injured body part’ based on the Injury Severity Score. The sample used for this histogram is restricted to those with at least a +/-2.5% difference in predicted probabilities. (TIF) [file pone.0268527.s006.tif]

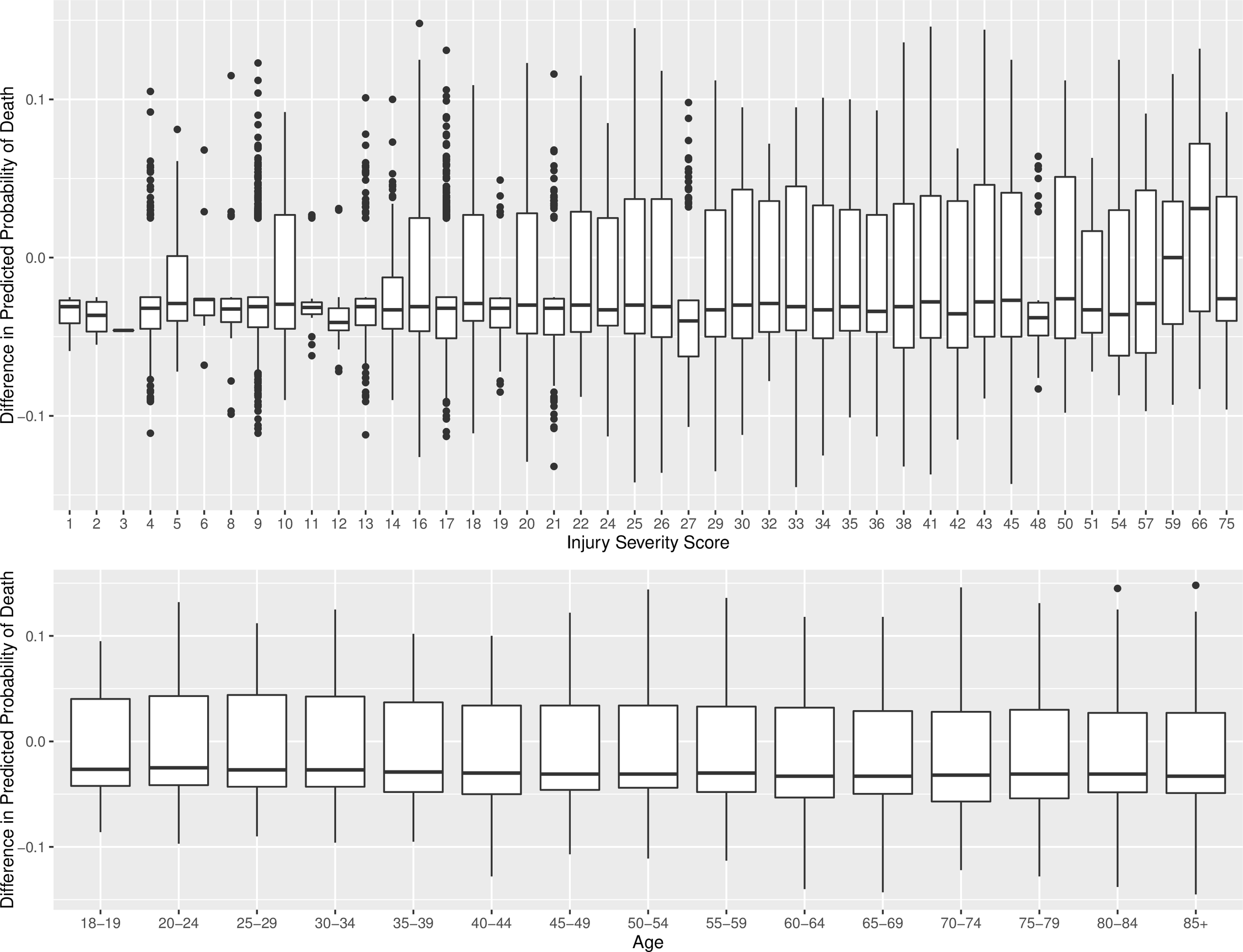

Supplement: S6 Fig — Median and IQR for differences in the predicted probability of 30-day all-cause mortality using a logistic regression model and all ancillary variables in combination with either the original or rescored version of the GCS, for binned age groups and Injury Severity Scores. The sample used for this histogram is restricted to those with at least a +/-2.5% difference in predicted probabilities. (TIF) [file pone.0268527.s007.tif]

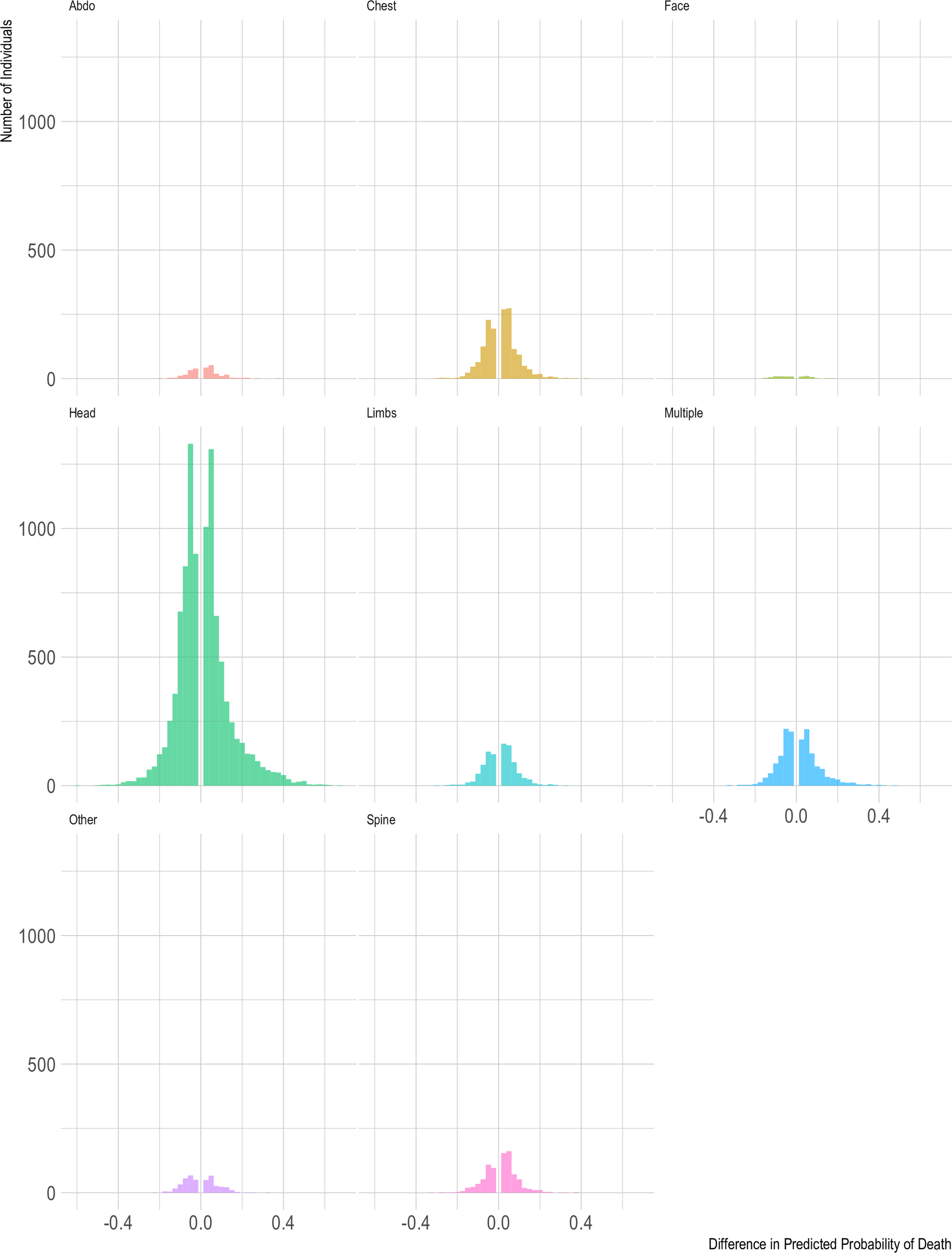

Supplement: S7 Fig — Differences in the predicted probability of 30-day all-cause mortality using a random forest model and all ancillary variables in combination with either the original or rescored version of the GCS, stratified by the ‘most severely injured body part’ based on the Injury Severity Score. The sample used for this histogram is restricted to those with at least a +/-2.5% difference in predicted probabilities. (TIF) [file pone.0268527.s008.tif]

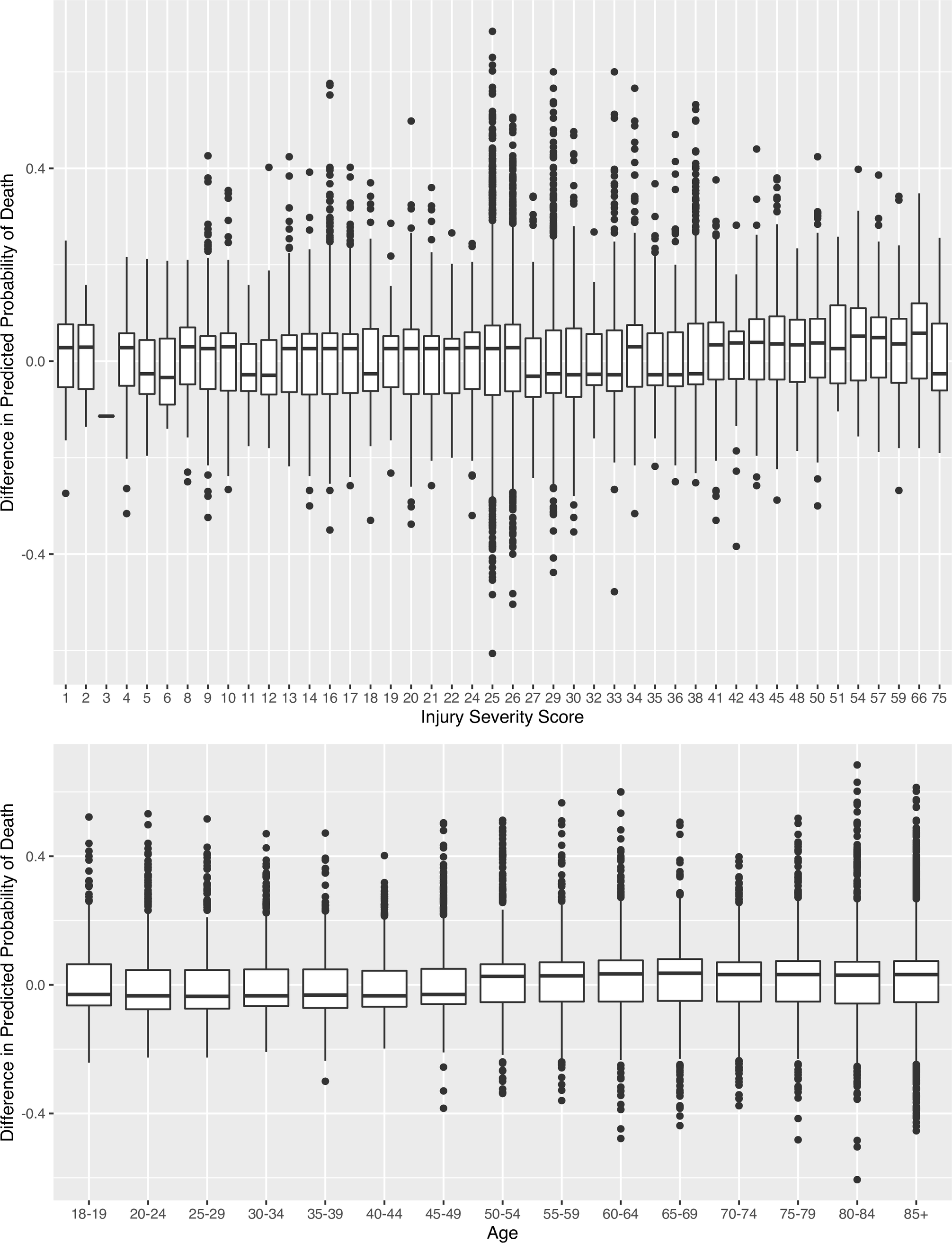

Supplement: S8 Fig — Median and IQR for differences in the predicted probability of 30-day all-cause mortality using a random forest model and all ancillary variables in combination with either the original or rescored version of the GCS, for binned age groups and Injury Severity Scores. The sample used for this histogram is restricted to those with at least a +/-2.5% difference in predicted probabilities. (TIF) [file pone.0268527.s009.tif]
